# El Niño Modoki can be mostly predicted more than 10 years ahead of time

**DOI:** 10.1038/s41598-021-97111-y

**Published:** 2021-09-09

**Authors:** X. San Liang, Fen Xu, Yineng Rong, Renhe Zhang, Xu Tang, Feng Zhang

**Affiliations:** 1 Department of Atmospheric and Oceanic Sciences, Institute of Atmospheric Sciences, Fudan University, No. 2005 Songhu Rd, Yangpu District, Shanghai, 200438 China; 2 Nanjing Center for Ocean-Atmosphere Dynamical Studies, Nanjing Institute of Meteorology, Nanjing, 210044 China; 3 Shanghai Qi Zhi Institute (Andrew C. Yao Institute), Shanghai, 200232 China; 4 IRDR ICoE on Risk Interconnectivity and Governance on Weather/Climate Extremes Impact and Public Health, Fudan University, Shanghai, 200438 China

**Keywords:** Climate sciences, Physical oceanography, Solar physics

## Abstract

The 2014–2015 “Monster”/“Super” El Niño failed to be predicted one year earlier due to the growing importance of a new type of El Niño, El Niño Modoki, which reportedly has much lower forecast skill with the classical models. In this study, we show that, so far as of today, this new El Niño actually can be mostly predicted at a lead time of more than 10 years. This is achieved through tracing the predictability source with an information flow-based causality analysis, which has been rigorously established from first principles during the past 16 years (e.g., Liang in Phys Rev E 94:052201, 2016). We show that the information flowing from the solar activity 45 years ago to the sea surface temperature results in a causal structure resembling the El Niño Modoki mode. Based on this, a multidimensional system is constructed out of the sunspot number series with time delays of 22–50 years. The first 25 principal components are then taken as the predictors to fulfill the prediction, which through causal AI based on the Liang–Kleeman information flow reproduces rather accurately the events thus far 12 years in advance.

## Introduction

El Niño prediction has become a routine practice in operational centers all over the world because it helps make decisions in many sectors of our society such as agriculture, hydrology, health, energy, to name a few. Recently, however, a number of projections fell off the mark. For example, in 2014, the initially projected “Monster El Niño” did not show up as expected; in the meantime, almost no model successfully predicted the 2015 super El Niño at a 1-year lead time^1^. Now it has been gradually clear that there are different types of El Niño; particularly, there exists a new type that warms the Pacific mainly in the center, and tends to be more unpredictable than the traditional El Niño with the present coupled models^2^. This phenomenon, which has caught much attention recently^3–7^ (c.f.^8^ for an earlier account), has been termed El Niño Modoki^6^, Central-Pacific (CP) type El Niño^5^, Date Line El Niño^4^, Warm Pool El Niño^7^, etc.; see^9^ for a review. El Niño Modoki has left climate imprints which are distinctly different from that caused by the canonical El Niño. For example, during El Niño Modoki episodes, the western coast region of the United States suffers from severe drought, whereas during canonical El Niño periods it is usually wet^10^; in the western Pacific, opposite impacts have also been realized for the tropical cyclone activities during the two types of El Niño periods. El Niño Modoki can reduce more effectively the Indian monsoon rainfall, exerting influences on the rainfall in Australia and southern China in a way different from that canonical El Niño does; also notably, during the 2009 El Niño Modoki, a stationary anticyclone is induced outside Antarctica, causing the melting the ice^1,11^.

El Niño Modoki prediction is difficult because its generating mechanisms are still largely unknown. Without a correct and complete attribution, the dynamical models may not have all the adequate dynamics embedded (e.g.,^12^). Though recently it is reported to correspond to one of the two most unstable modes^13^ for the Zebiak–Cane model^14^, the excitation of the mode, if true, remains elusive. It has been argued that the central Pacific warming is due to ocean advection^7^, wintertime midlatitude atmospheric variations^15^, wind-induced thermocline variations^6^, westerly wind bursts^16^, to name a few. But these proposed mechanisms are yet to be verified. One approach to verification is to assess the importance of each factor through sensitivity experiments with numerical models. The difficulty here is, a numerical climate system may involve many components with parameters yet to be determined empirically, let alone the highly nonlinear components, say, the fluid earth subsystem, may be intrinsically uncertain. Besides, for a phenomenon with physics largely unknown, whether the model setup is dynamically consistent is always a concern. For El Niño Modoki, it has been reported that the present coupled models tend to have lower forecast skill for El Niño Modoki than that for canonical El Niño^2^, probably due to the fact that, during El Niño Modoki, the atmosphere and ocean fail to connect. For all the above, without a correct attribution, it is not surprising to see that the 2014–2015 forecasts fell off the mark—essentially no model predicted the 2015 El Niño at a 1-year lead time (cf.^17^).

Rather than based on dynamical models, an alternative approach relies heavily on observations and gradually becomes popular as data accumulate. This practice, commonly known as statistical forecast in climate science (e.g.,^18^) actually appears in a similar fashion in many other disciplines, and now has evolved into a new science, namely, data science. Data science is growing rapidly during the past few years, one important topic being predictability. Recently it has been found that predictability can be transferred between dynamical events, and the transfer of predictability can be rigorously derived from first principles in physics^19–21^. The finding is expected to help unravel the origin(s) of predictability for El Niño Modoki, and hence guide its prediction. In this study, we introduce this systematically developed theory, which was in fact born from atmosphere-ocean science, and, after numerous experiments, show that El Niño Modoki actually can be mostly predicted at a lead time of 13 years or over, in contrast to the canonical El Niño forecasts. We emphasize that it is NOT our intention to do dynamical attributions here. We just present a fact on accomplished predictions. There is still a long way to go in pursuit of the dynamical processes underlying the new type of El Niño.

### Seeking predictor(s) for El Niño Modoki with information flow analysis

The major research methodology is based on a recently rigorously developed theory of information flow^20^, a fundamental notion in physics which has applications in a wide variety of scientific disciplines. Its importance lies beyond the literal meaning in that it implies causation, transfer of predictability, among others. Recently, it has just been found to be derivable from first principles (rather than axiomatically proposed)^20^, initially motivated by the predictability study in atmosphere-ocean science^19^. Since its birth it has been validated with many benchmark dynamical systems (cf.^20^), and has been successfully applied to different disciplines such as earth system science, neuroscience, quantitative finance, etc. (cf.^22–24^). Refer to the “Method” section for a brief introduction.

We now apply the information flow analysis to find where the predictability of El Niño Modoki is from. Based on this it is henceforth possible to select covariate(s) for the prediction. Covariate selection, also known as sparsity identification, is performed before parameter estimation. It arises in all kinds of engineering applications such as helicopter control^25^ and other mechanical system modeling^26^, as well as in climate science. We particularly need to use the formula (6) or its two-dimensional (2D) version (7) as shown in the “Method” section. The data used are referred to the Data Availability Section.

At the first step we sort of use brute force: compute the information flows from the index series of some prospect driver to the sea surface temperature (SST) at each grid point of the tropical Pacific. Because of its quantitative nature, the resulting distribution of information flow will form a structure which we will refer to “causal structure” henceforth. While it may be within expectation that information flow may exist (different from zero) for the SST at some grid points, it is not coincidental if the flows at all grid points organize into a certain pattern/structure. (This is similar to how teleconnection patterns are identified^27^.) We then check whether the resulting causal structure resembles the El Niño Modoki pattern, say, the pattern in the Fig. 2b of^6^. We have tried many indices (e.g. those of Indian Ocean Dipole, Pacific Decadal Oscillation, North Atlantic, Atlantic Multi-decadal Oscillation, to name but a few), and have found that the series of sunspot numbers (SSN) is the very one. In the following we present the algorithm and results.

We first use the 2D version of Eq. (6) (cf. (7)) to do a rough estimate for the information flow from SSN to the SST at each grid point, given the time series as described in the preceding section. This is the practice how teleconnection patterns are identified using correlation analysis, but here the computed is information flow. (Different from the symmetric correlation, here the information flow in the opposite direction is by computation insignificant, just as expected.) We then form delayed time series for SSN, given time delays $n=1,2,\ldots$, and repeat the above step. For convenience, denote an SSN series with delay *n* by SSN($-n$). We find that the resulting information flow is not significant at a 90% confidence level, or has a structure bearing no resemblance to the desired pattern, until *n* approaches 45 years; see Supplementary Fig. S1, for a number of examples. Here the data we are using are the SST from 01/1980 through 12/2017 (SST are most reliable after 1979), and the SSN data are correspondingly from 01/1935 through 12/1972. Note that using the time delayed coordinates we may reconstruct a dynamical system which is topologically equivalent to the one that originally generates the time series, thanks to Takens’ embedding theorem^28^. (A topologically equivalent reconstruction is in general difficult, as here we do not know the dimension of the manifold underlying the solar activity.) Here, based on the above rough estimates, we choose to form a dynamical system with components corresponding to the SST series and the time-delayed series SSN($-n$) with *n* ranging from 22 to 50 years. Note one is advised not to choose two delays too close. SSN is dominated by low-frequency processes; two close series thus-formed may not be independent enough to span a subspace, and hence may lead to singularity. Here we choose SSN series at delays of 22–50 years every 5 years or 60 steps. We have tried many other sampling intervals and found this is the best. From the thus-generated multivariate series, we then compute the information flow from SSN($-45$ years) to the Pacific SST, using formula (6).

A note on the embedding coordinates. In general, it is impossible to reconstruct exactly the original dynamical system from the embedding coordinates; it is just a topologically equivalent one at most. That also implies that the reconstruction is not unique. Other choices with delayed time series may serve the purpose as well, provided that they are independent of other coordinates; see^29^ for empirical methods for time delay choosing. Fortunately, this does not make a problem for our causal inference, thanks to a property of the information flow *ab initio*, which asserts that

- The information flow between two coordinates of a system stays invariant upon arbitrary nonlinear transformation of the remaining coordinates.

More details are referred to the “Method” section. This remarkable property implies that the embedding coordinates do not matter in evaluating the information flow from SSN to SST; what matters is the number of the coordinates, i.e., the dimension, of the system, which makes a topological invariant. There exist empirical ways to determine the dimension of a system, e.g., those as shown in^29^. Here we choose by adding new coordinates and examining the information flow; if it does not change much, the process stops and the dimension is hence determined. For this problem, as shown above, the SSN series at delays of 22–50 years every 5 years are chosen. This results in six auxiliary coordinates, which together with the SST and SSN($-45$ years) series make a system of dimension 8.

**Figure 1:**
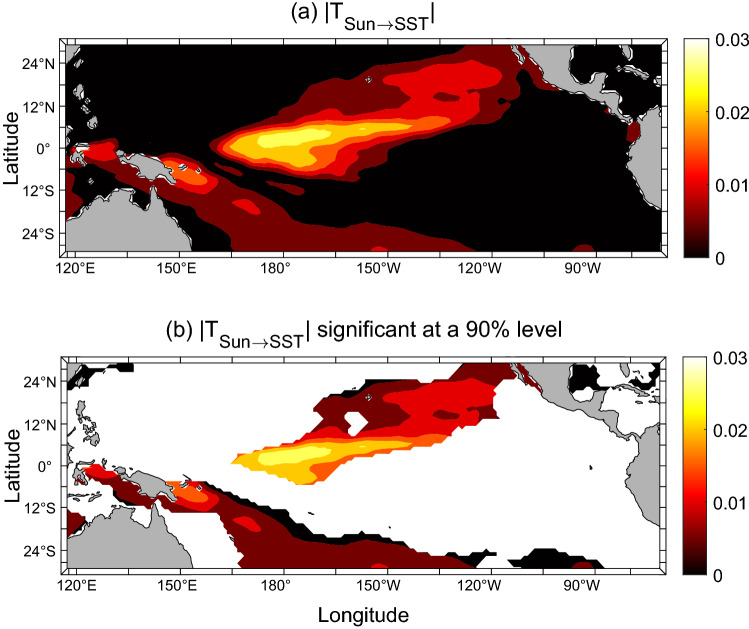
(**a**) The absolute information flow from the delayed (by 45 years) series of sunspot numbers to those of the sea surface temperature in the Pacific Ocean (in nats/month). The pattern resembles very much the El Niño Modoki mode as shown in the Fig. 2b of^6^. Here information flow means the transfer of predictability from one series to another. It is computed with Eq. (6) in “Method”. (**b**) As (**a**), but only the information flow significant at a 90% confidence level is shown. (Figure generated with MATLAB, Version 6.5. http://www.mathworks.com/).

The spatial distribution of the absolute information flow, or “causal structure” as will be referred to henceforth, from SSN($-45$ years) to the Pacific SST is shown in Fig. 1.

Remarkably, the causal structure in Fig. 1a is very similar to the El Niño Modoki pattern, as given by, say, Ashok et al.^6^. (Please see their Fig. 2b.) That is to say, the information flow from SSN to the Pacific SST does form a spatially coherent structure resembling to El Niño Modoki.

To see whether the computed causal structure or causal pattern is statistically significant, significance test is performed using the techniques as described in the “Method” section. The computationally simple one is with the Fisher information matrix; by it the causal structure is significant at a 90% confidence level, as shown in Fig. 1b. More robust testing requires that surrogate data be generated with an autoregressive model. Details are referred to the “Method” section, and the results are presented in Supplementary Figs. S2 and S3. As can be seen, the causal structure is significant at a level of 99%. We have tried 100 and 200 surrogates for each series and the results are similar.

Comparing to the rough estimate in the bivariate case (see Supplementary Fig. S1d), Fig. 1a has a horseshoe structure pronounced in the upper part, plus a weak branch in the Southern Hemisphere. This is just as the El Niño Modoki structure as obtained by Ashok et al.^6^ (see their Fig. 2b). This indicates that the bivariate information flow analysis provides a good initial guess, but the result is yet to be rectified due to the potentially incorrect dimensionality.

The information flows with other time delays have also been computed. Shown in Supplementary Fig. S4, are a number of examples. As can be seen, except those around 45 years (about 44–46 years, to be precise), other delays either yield insignificant information flows or do not result in the desired causal pattern.

Since we are about to predict, it is desirable to perform the causality analysis in a forecast fashion, i.e., to pretend that the SST, hence the El Niño Modoki Index (EMI) that measures the strength of the event, over the forecast period be unavailable. We have computed the information flow with different years of availability. Shown in Supplementary Fig. S5 is the information flow as that in Fig. 1, but with SST data available until the year of 2005. Obviously, the causal pattern is still there and significant, and, remarkably, it appears even more enhanced, probably due to the frequent occurrences of the event during that period.

As information flow tells the transfer of predictability, SSN forms a natural predictor for El Niño Modoki. This will be further confirmed in the next section.

### Prediction of El Niño Modoki

The remarkable causal pattern in Fig. 1 implies that it may be possible to make forecasts of El Niño Modoki many years ahead of time. Based on it we hence conjecture that the lagged SSN series be the desired covariate. Of course, due to the short observation period, the data needed for parametric estimation are rather limited—this is always a problem for climate projection.

**Figure 2:**
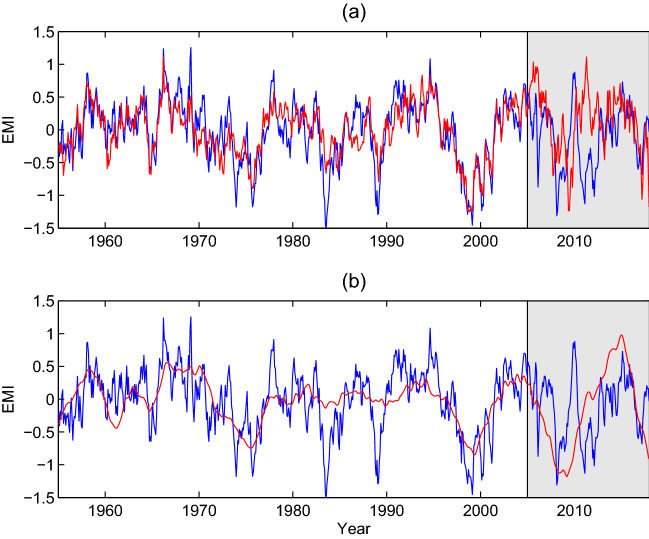
Prediction of the El Niño Modoki index based solely on the sunspot numbers 22–50 years ago with a simple linear regression model. (**a**) The SSN series are bandpass filtered with the annual signals and the signals longer than 11 years removed. (**b**) Only 25 first principal components are used as inputs. The shaded period is for prediction, and the predicted index is in red. (Figure generated with MATLAB, Version 6.5. http://www.mathworks.com/).

Let us start with a simple linear regression model for the prediction of El Niño Modoki Index (EMI) from SSN. Build the model using the EMI data from 1975–2005 ($30\times 12=600$ equations), leaving the remaining years after 2005 for prediction. For each EMI, the model inputs are the SSNs at lead times of 50 years through 22 years (336 in total), guided by the causality analysis in the preceding section. For best result, the annual signals and the signals longer than 11 years are filtered from the SSN series. The filtering is fulfilled through wavelet analysis with the orthonormal spline wavelets built in^30^. Since it is required that the series length be a power of 2, we choose a time range for the monthly SSN series from May 1847 to December 2017, depending on the availability of the data when this research was initialized. This totals 170 years and 8 months (170.67 years), or $2^{11}=2048$ time steps. The upper bound scale level for the wavelet analysis is set 8, which gives a lower period of $2^{-8} \times 170.67 = 0.67$ years; the lower bound scale level is chosen to be 4, resulting an upper period of $2^{-4} \times 170.67 = 10.67$ years. In doing this the seasonal cycle and the interdecadal variabilities are effectively removed (Supplementary Fig. S6). With the pretreated SSN series the prediction is launched, and the result is shown in Fig. 2a.

We would not say that the prediction in Fig. 2a with such a simple linear model is successful, but the 2015/2016 event is clearly seen. The strong 2009 event is not correct; it has a phase error. The general trend between 2005 and 2018 seems to be fine. Beyond 2018 it becomes off the mark.

It would be of some use to see how the simple linear prediction may depend on some factors, though it cannot be said successful and hence a quantitative investigation does not make sense. We hope to learn some experience for the machine learning to be performed soon. First, we check the effect of filtering. As shown in Supplementary Fig. S7, without filtering, the result is much noisier, the 2015/2016 event is too strong, and the strong 2009/2010 event completely disappears; with low-pass filtering (only the signals below a year are filtered), the result is similar, only with the noise reduced and the 2015/2016 event better.

Second, we perform an empirical orthogonal function (EOF) analysis for the 336 time-delayed SSN series, which forms a column vector with 336 entries at each time step: [${\text{SSN}}(n-600), \text{SSN}(n-599),\ldots , {\text{SSN}}(n-265)$]$^T$. The variance for each EOF mode is plotted in Supplementary Fig. S8. From it the first 8, 25, and 50 principal components (PCs) approximately account for 84%, 89%, and 92% of the total variances, respectively. Some examples of the EOF modes are plotted in Supplementary Fig. S9. Now use the first 25 PCs as inputs and repeat the above process to make the prediction. The result is shown in Fig. 2b. Obviously, during the 12 years of prediction from 2005 to 2017, except for the strong 2009/2010 event, the others are generally fine. Projections with other numbers of PCs have also been conducted. Shown in Supplementary Fig. S10 are examples with 8 and 50 PCs, respectively. It is particularly interesting to see that, except for the 2009/2010 event, the case with 8 PCs already captures the major trend of the EMI evolution. Recall that, in performing the information flow analysis, we have found that the reconstructed dynamical system approximately has a dimension of 8. Here the prediction agrees with the dimension inference.

**Figure 3:**
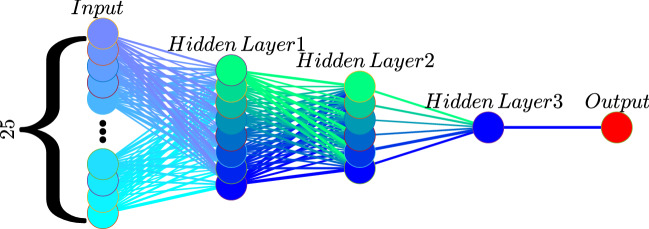
The neural network architecture used in this study, which is made of an input layer, an output layer, and three hidden layers. The symbols are the same as those in (1). The matrices/vectors $\mathbf{w}_i$ and $\mathbf{b}_i$ ($i=1,2,3,4$) are obtained through training. To predict the El Niño Modoki Index (EMI) at month/step *n*, ${\text{EMI}}(n)$, find the first 25 principal components of [${\text{SSN}}(n-600), {\text{SSN}}(n-599),\ldots , {\text{SSN}}(n-265)$]$^T$, a column vector with 336 entries, and form the input vector $\mathbf{x}$. The final output *y* is the predicted ${\text{EMI}}(n)$. Iterating on *n*, we can get a prediction of EMI for any target time interval. (Figure generated with MATLAB, Version 6.5. http://www.mathworks.com/).

Because of the encouraging linear model result, it is desirable to achieve a better prediction using more sophisticated tools from artificial intelligence (AI)/deep learning, e.g., the back propagation (BP) neural network algorithm^31^. (At the preparation of the original version of this manuscript, we noticed that recently there have been applications of other machine learning methods to El Niño forecasts, e.g.,^32^.) Three hidden layers are used for the BP neural network forecast. Again, a major issue here is the short observational period which may prevent from an appropriate training with many weights. We hence use only 8, 6, and 1 neurons for the first, second, and third hidden layers, respectively, as schematized in Fig. 3. This architecture will be justified soon. To predict an EMI at step (month) *n*, written *y*, a $336\times 1$ vector $\mathbf{x}$ is formed with SSN data at steps (months) $n-600, n-599,\ldots , n-265$, the same as the case with lead times of 50–22 years for the above simple linear regression model. (Note here the “predictions” include those on the validation set.) But even with the modest number of parameters, the data are still not enough. To maximize the use of the very limited data, we choose the first 25 principal components (PCs) that account for 90% of the total variance. We hence train the model with these 25 inputs (rather than 336 inputs); that is to say, $\mathbf{x}$ now is a $25\times 1$ vector, which greatly reduces the number of parameters to train. (See below in Eq. (1): The $\mathbf{w}_1$ is reduced from a $8\times 336$ matrix to a $8\times 25$ matrix.) Once this is done, $\mathbf{x}$ is input into the following equation to arrive at the prediction of *y*:

1 $$\begin{aligned} y = {\mathbf{w}_4}\ {\text{tansig}} ({\mathbf{w}_3}\ {\text{tansig}} (\mathbf{w}_2\ {\text{tansig}}(\mathbf{w}_1\ \mathbf{x} + \mathbf{b}_1) + \mathbf{b}_2) + \mathbf{b}_3) + \mathbf{b}_4, \end{aligned}$$

where the matrices/vectors of parameters $\mathbf{w}_1$ ($8\times 25$ matrix), $\mathbf{w}_2$ ($6\times 8$ matrix), $\mathbf{w}_3$ ($1\times 6$ row vector), $\mathbf{w}_4$ ($1\times 1$ matrix—a scalar), $\mathbf{b}_1$ ($8\times 1$ vector), $\mathbf{b}_2$ ($6\times 1$ vector), $b_3$ (scalar) and $b_4$ (scalar) are obtained through training with trainbr, a network training function that updates these weights through the Levenberg-Marquardt optimization. The training stops when any of the following conditions occurs: (1) maximum number of epochs reached (set to be 1000); (2) performance minimized to the goal (set to be $1\times 10^{-5}$); (3) performance gradient falling below min_grad (set to be 20); (4) the adaptive value in the Bayesian regularization, $\mu$, exceeding $\mu _{\max }$ (set to be $1\times 10^5$). See^33^ for details. An illustrative explanation is seen in Fig. 3. As has been argued (e.g.^34^) that the number of hidden neurons should be kept fewer than 2/3 the size of the input and output layers, i.e., $\frac{2}{3}\times (25+1)=17$ here. With this architecture we have 15 neurons in total, meeting the requirement.

To predict the EMI at month/step *n*, ${\text{EMI}}(n)$, organize ${\text{SSN}}(n-600), {\text{SSN}}(n-599), \ldots , {\text{SSN}}(n-265)$, that is, the SSNs lagged by 50 through 22 years, into a $336\times 1$ vector. Project it onto the first 25 EOFs as obtained above, and obtain $\mathbf{x}$, a vector with 25 entries. Input $\mathbf{x}$ into Eq. (1). The final output *y* is the predicted ${\text{EMI}}(n)$. Iterating on *n*, we can get a prediction of EMI for any target time interval.

The 5-year data prior to the starting step of prediction are used for validation. In this study, we start off predicting EMI at January 2008. We hence take the data over the period from January 2003 through December 2007 to form the validation set, and the data until December 2002 to train the model. The validation set is kind of short but we have to live with it, otherwise there would be insufficient data for the training. We have also tried different intervals of the same length for validation. The results are poor, acceptable, and successful respectively for those before 1980, those between 1985 through 1995, and those after 1995. This makes sense, for atmosphere/ocean/climate problems are notoriously predictability limited as time moves on (the arrow of time; more below on cross-validation), and hence the most recent time interval(s) should be chosen to form the validation set (e.g.,^35,36^). To test whether the model performance is sensitive to the validation sample size, we have utilized the “new” data from 2008 through 2020 and make predictions into the future from now. Validation sets of different length have been chosen on the interval 2003–2020: 2003–2009, 2010–2020, 2014–2020, and the results are essentially the same.

**Figure 4:**
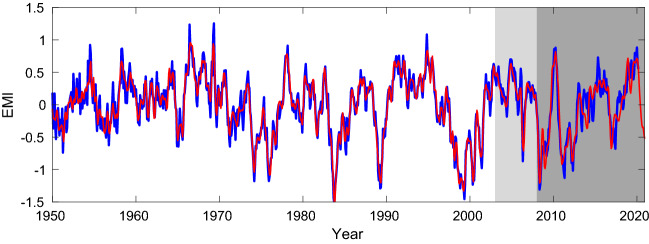
Prediction of the El Niño Modoki index based solely on the sunspot numbers 22–50 years ago with the back propagation neural network algorithm. The EMI is in blue, while the predicted index is in red. Lightly shaded is the period over which the validation set is formed, and the period with dark shading is for prediction. (Figure generated with MATLAB, Version 6.5. http://www.mathworks.com/).

10,000 runs have been performed, and we pick the one that minimizes the mean square error (MSE) over the validation set. This is plotted in Fig. 4. Remarkably, the 12-year long index has been forecast with high skill (corr. coeff. = 0.91). Particularly, the strong 2009/2010 event is well predicted, so is the 2019/2020 event. The 2014/2016 event, which has made the El Niño forecasts off the mark in 2014–2015, appears a little weaker but also looks fine by trend.

**Figure 5:**
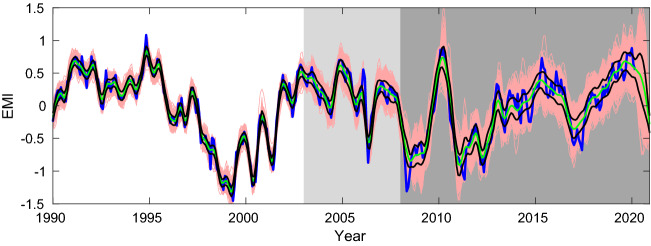
The 1000 predictions showing the spread of the ensemble. Overlaid are the observed EMI (blue), the mean of the realizations (cyan), and the mean plus and minus the standard deviation (black). The light shading marks the period for validation, while the darker shading marks the prediction period. (Figure generated with MATLAB, Version 6.5. http://www.mathworks.com/).

Out of the 10,000 runs we select the top 10% with lowest MSE over the validation set (January 2003–December 2007) to form an ensemble of predictions for the 12 years of EMI (January 2008 till now). The spread, the mean, and the standard deviation of the ensemble are shown in Fig. 5. Also overlaid is the observed EMI (blue). As can be seen, the uncertainty is rather limited for a climate projection.

**Figure 6:**
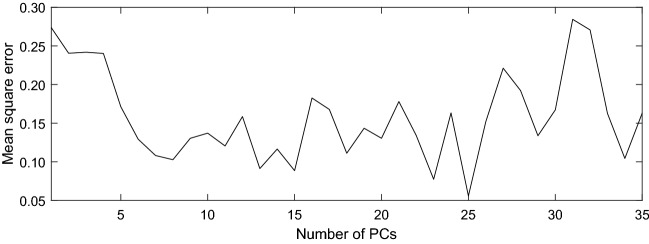
Mean square error for the neural network prediction as a function of number of principal components. (Figure generated with MATLAB, Version 6.5. http://www.mathworks.com/).

We have examined how the prediction performance may vary with the choices of PCs, the number of neurons, and the lags. The PCs of a vector are obtained by projecting it onto the EOF modes (described above), which possess variances and structures as plotted in Supplementary Figs. S8 and S9, respectively. Shown in Fig. 6 is the MSE as a function of the number of PCs. Obviously with 25 PCs the MSE is optimized, and that is what we have chosen for the network architecture. But if one takes a closer look, the MSE does not decrease much beyond 8. Recall that, in performing the causality analysis, we have seen that the reconstructed dynamical system approximately has a dimension 8. Fig. 6 hence provides a verification of the dimension inference.

The EOF modes associated with these 8 PCs are plotted in Supplementary Fig. S9. As can be seen, except for the 11-year process, among others, the 5-year variability is essential. This is, again, consistent with what we have done in forming the embedding coordinates: select the delayed series every 5 years.

**Figure 7:**
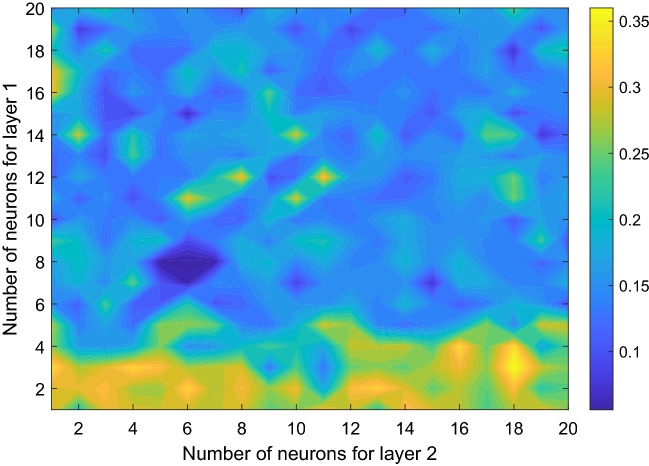
Mean square error as function of number of neurons. (Figure generated with MATLAB, Version 6.5. http://www.mathworks.com/).

The dependence of the model performance on the number of neurons has also been investigated. We choose 1 neuron for layer 3, leaving the numbers of layers 1 and 2 for tuning. The result is contoured in Fig. 7. As can be clearly seen, a minimum of MSE appears at (8,6), i.e., when the numbers of hidden layer 1 and layer 2 are, respectively, 8 and 6. This is what we have chosen to launch the standard prediction.

**Figure 8:**
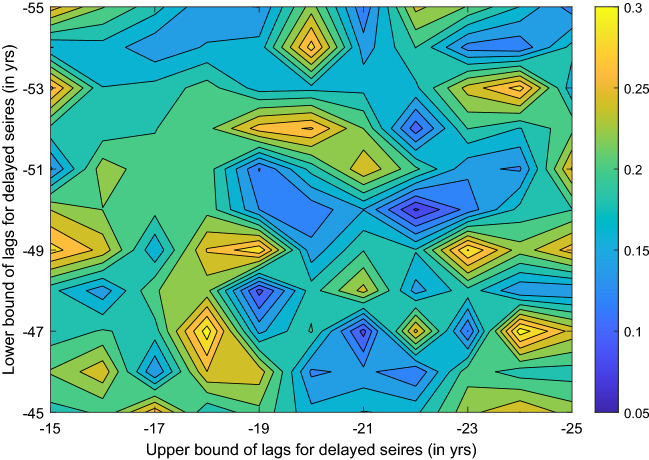
Mean square error as function of lags. (Figure generated with MATLAB Version R2020b. http://www.mathworks.com/).

Also studied is the influence of the lags for the delayed series. In Fig. 8 the MSE is shown as function of the lower and upper bounds of the delays (in years). From the figure there are a variety of local minima, but the one at (22, 50) is the smallest one. That is to say, the series with delays from 22 to 50 years make the optimal embedding coordinates, and this is just what we are choosing.

We have tried to make hindcasts from the limited data to cross-validate the forecasts. (Many thanks to an anonymous reviewer for the input.) From the whole history 1920–2020 we pick a 5-year interval, say [1920, 1924], as the validation period, and use the rest data to train the model. Then, swap the interval until all the 5-year intervals throughout have been utilized. The “forecasts” for all the validation periods are then pieced together to form a plot (Supplementary Fig. S11, upper panel), which allows one to see the possible time dependence of the forecast skill. From it we see that the “forecasts” after 1980 are good, but those before are not, though the trend looks fine. This could be either due to the poor quality of observation before 1980 or due to the arrow of time as mentioned above. To illustrate, consider a simple partial differential equation that mimics a linearized “one-dimensional atmospheric flow”

$$\begin{aligned} \frac{\partial u}{\partial t} + a\frac{\partial u}{\partial x} = \frac{1}{2} \nu \frac{\partial ^2 u}{\partial x^2}, \end{aligned}$$

for $-\infty<x<\infty$, $t\ge 0$. Let, for example, $a=1$, $\nu =0.1$, and initially ($t=0$) an impulse is placed at $x=0$. The solution can be found analytically. If we take measurements at $x_1=0$ and $x_2=2$, two time series $u_1(t) = u(0,t)$ and $u_2(t)=u(2,t)$ are obtained. Note obviously $u_1$ is causal to $u_2$, but not the other way around. We hence can use the past history of $u_1$ to predict the future of $u_2$, with the above deep learning technique. Since the diffusion process is irreversible, it is generally impossible to use the future data of $u_1$ to “predict” the past of $u_2$. $u_2(1)$, This is a simple example showing the “arrow of time” in the atmosphere–ocean processes.

We have tried different validation periods, e.g., a 10-year period and a 20-year period, for the cross-validation. The result with the 20-year period is shown in Supplementary Fig. S11 (lower panel), which is generally similar to the one with the 5-year period.

It is known that machine learning suffers from the problem of reproducibility^37^. But in this study, as we have shown above, the prediction guided by the information flow analysis is fairly robust, with uncertainty acceptably small for climate projection. For all that account, the El Niño Modoki events so far are mostly predictable at a lead time of more than 10 years.

### Causality evolution and prediction into the future

We want to emphasize again that it is NOT our intention to make dynamical attribution, and hence the above trained model, which is based on the data of the past century, may not be universally applicable. Nonetheless, with the model it is tempting to make forecasts into the future. This is however risky, due to some unforeseen reasons (e.g., the causality problem as illustrated below), although the prediction made early in 2018 (when the original manuscript was prepared) agrees with the observation so far as of today. Here the best 1000 out of the 10,000 forecasts are plotted in Supplementary Fig. S12a (“best” in the sense of minimal bias over the validation period 01/2003–12/2007.) Correspondingly the RMS is as shown in Supplementary Fig. S12b. As can be seen from the spread and the RMS, the uncertainty becomes large after 2020, and predictability is soon lost after that. Another observation is that the RMS varies decade by decade. The decadal variation of predictability with the system is another topic that deserves further study.

A reason for this kind of phenomenon is that causality has changed, and hence predictability is being lost. In general causality varies with time; estimated in Fig. 1 is an overall one over the entire period in question. Varying causality has been examined in many different studies (e.g.,^24,38^). Something similar happens with El Niño cycle’s causality, as discovered in^39^. In order to see how causality evolves, we need to choose a time window of some length, compute the information flow on that window, slide it forward and repeat the computation. The resulting information flow on a window can be taken as that at the center of that window. For this purpose, the window size should be as small as possible, so as to arrive at a causality local enough. But it must also be long enough to have “sufficient” data. Here for a rough estimate, we choose it to be one solar cycle. Considering its short length, we can only try the bivariate formula (7). The results are plotted in Supplementary Fig. S13. As displayed, the causal pattern from SSN to the SST lagged by 45 years looks good in 1960s, 1970s, 1990s, 2000s, and is not good in 1950s, 1980s. In the most recent decade, 2010–2020, the pattern has changed to something else, with a strong center appearing in the equatorial region north of Australia, as the one in 1950s and 1980s. Here we emphasize that this is just a rough estimate, since the system does not need to be 2D, and, besides, the series for just one solar cycle are generally not long enough. But the results do give us a hint on the reduction in causality, which leads to the predictability loss as observed above.

**Figure 9:**
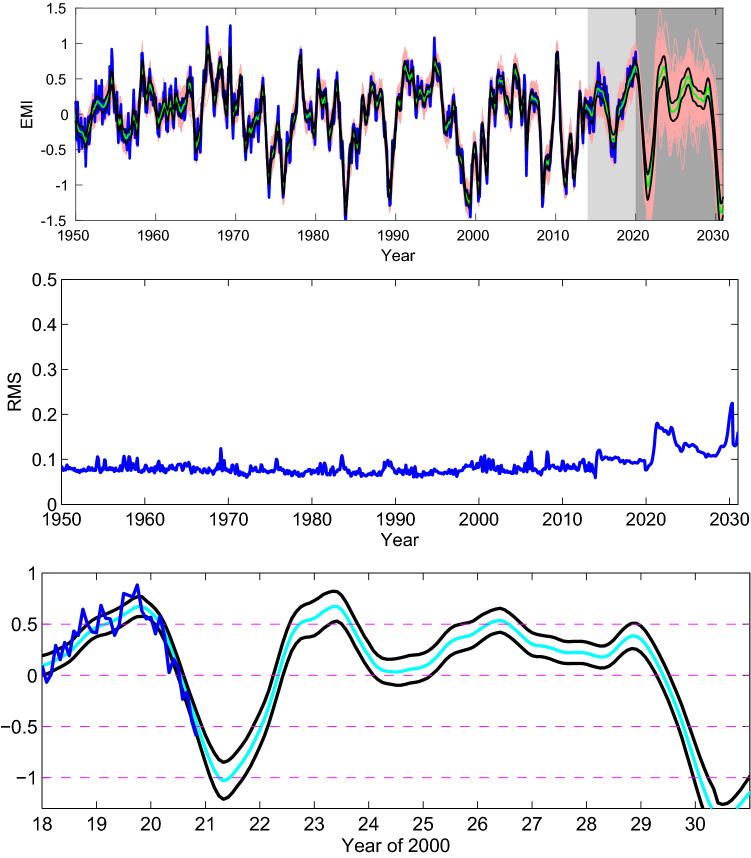
Top: As Fig. 5, but using a model based on series with delays from 10 through 43 years, with the time intervals forming the training set and validation set being, respectively, 01/1920–12/2013 and 01/2014–02/2020. Middle: The corresponding standard deviation around the ensemble mean. Bottom: A close-up of the prediction over 2018–2030. (Figure generated with MATLAB, Version 6.5. http://www.mathworks.com/).

The loss of predictability may be embedded in other time delayed series. We hence try a 2D causality analysis with other lags for the interval 2010–2020. Interestingly, the familiar pattern reappears for many lags between 10 and 43 years, as shown in Supplementary Fig. S14. Note again these information flows are for reference only, though the major features are significant (not shown). This, however, guides our choice of delays in building the model. In order to utilize the observations over the recent decade, we hence re-train the model using these new delayed series, with all other settings as before. The prediction together with its RMS (middle panel) is shown in Fig. 9. For clarity, the ensemble mean and standard deviation are zoomed in on the interval 2018–2030 (lower panel). By the RMS distribution, the prediction after 2030 with this model should be interpreted with caution because of the large uncertainty thenafter.

Identified from the figure are four local maxima since 2020, plus two cold events. These events are tabulated in Table 1. The confidence interval is at a level of 90% (standard deviation multiplied by 1.65). From the table it is likely that an El Niño Modoki will take place in the winter (DJF) of 2022–2023. Also there could be an El Niño Modoki in the winter of 2025–2026 (marginal). Very probably La Niña Modoki events will occur in the winters of 2020–2021 and 2030–2031. Of course the projected event around 2030 is more than 10 years ahead; caution should be used here. These are what we can tell so far. As we have claimed before, the purpose of this study is just to report a fact about the accomplished predictions for the El Niño Modoki events during the past century; for real-time forecasts, a huge amount of effort should be invested in an operational mode, and that is certainly beyond the scope of this short report.

**Table 1:** A table of the timings and strengths of the possible extreme El Niño Modoki events in 2020s. The confidence interval is at the 90% level.

El Niño Modoki	DJF 19–20	DJF 22–23	DJF 25–26	DJF 28–29
EMI	$0.67\pm 0.16$	$0.67\pm 0.24$	$0.54\pm 0.19$	$0.39\pm 0.20$

La Niña Modoki	DJF 20–21	DJF 29–30		
EMI	$-1.03 \pm 0.30$	$-1.30 \pm 0.22$		

## Concluding remarks

The rigorously developed theory of information flow^20,40^, and hence causality, during the past decade provides a natural way for one to seek predictor(s) for a dynamical phenomenon, and this forms the basis of causal AI. In this study, it is found that the delayed causal pattern from SSN to the Pacific SST resembles very much the El Niño Modoki mode; the former hence can be used to predict the latter. Indeed, as detailed above, with all the observations we have had so far, the El Niño Modoki events so far as of today can be essentially predicted based solely on SSN at a lead time of 12 years or over. Particularly, the strong event in 2009/2010 and the elusive event during 2014–2016 have been well predicted (Fig. 2b). Moreover, the prediction of the events in the winters of 2019–2020 and 2020–2021 made early in 2018 (when the original manuscript was prepared) agrees well with the observation.

We, however, do NOT claim that El Niño Modoki is ultimately driven by solar activities. Indeed, as is found above, the causality is actually changing. It is NOT our intention in this study to investigate on the dynamical aspects of this climate mode. We just present an observational fact on fulfilled predictions. There is still a very long way to go in unraveling the dynamical origin(s) of El Niño Modoki. The success of atmosphere-ocean coupled models during the past decades confirms that the canonical El Niño is an intrinsic mode in the climate system; El Niño Modoki may be so also. Nonetheless, despite the long-standing controversy on the role of solar activity in climate change (see a review in^41^), there does exist evidence on the lagged response as identified here; the North Atlantic climate response is such an example^42^.

Nonetheless, one may wonder why there exists such a high predictability so far as of today. As commented by a reviewer, the results seem to suggest that “the variability in solar forcing is amplified and nonlinearly converted by the climate system to result, with a delay, in the evolution of the El Niño Modoki pattern,” and that, if there are first principle governing equations for El Niño Modoki, the related “SST initial condition is completely determined by the past SSN predictor.” These inspiring conjectures, among many others, need to be carefully addressed in future studies, in order to have a better understanding of the predictability as observed with the aid of our causality analysis.

**Figure 10:**
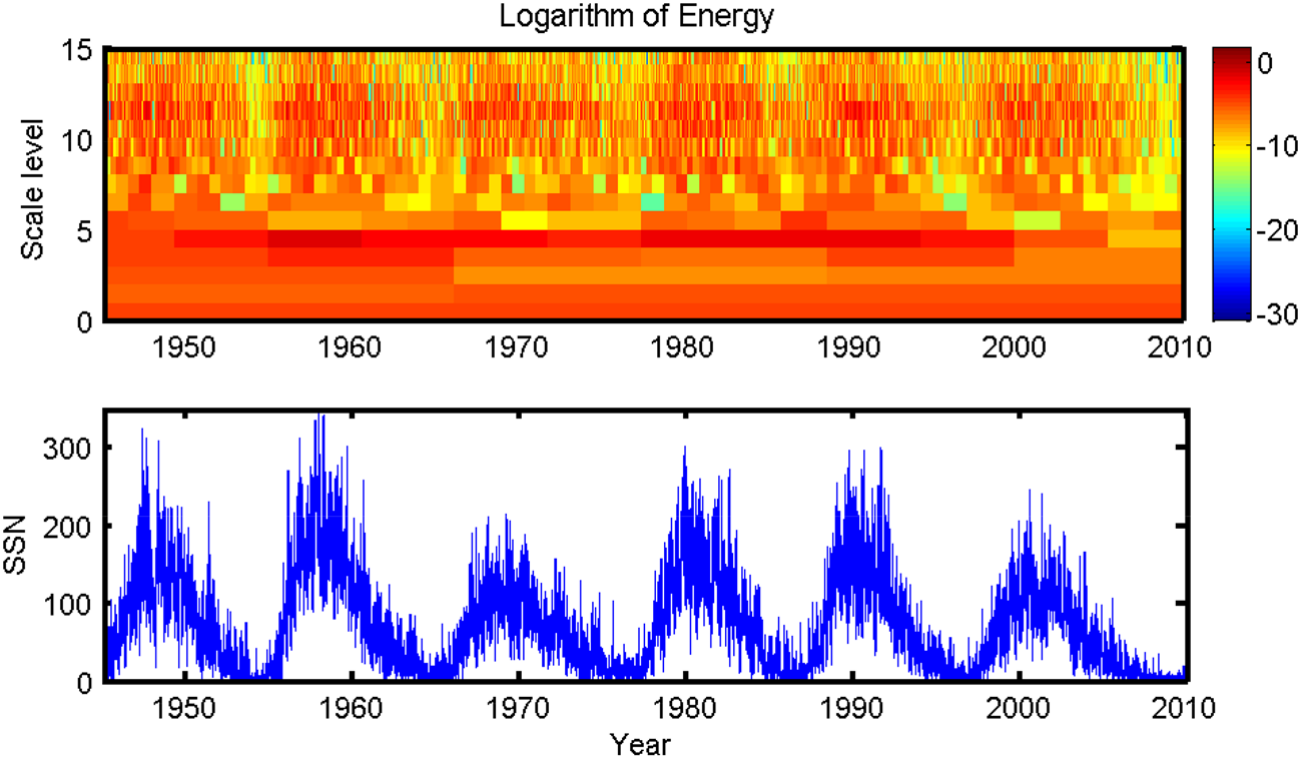
Daily series and orthonormal wavelet spectrum of the sunspot numbers based on the spline wavelets built in^30^. (Figure generated with MATLAB, Version 6.5. http://www.mathworks.com/).

As a final remark, it is interesting to see that the maximum information flow from the SSN lagged by 45 years to the Pacific SST agrees very well with frequent occurrences of El Niño Modoki during the period of 2000–2010: three of the four El Niño events are of this type (i.e., the 02/03, 04/05, 09/10 episodes). If we go back to 45 years ago, the sun is most active during the period of 1955–1965 (Fig. 10) Particularly, in the wavelet spectrum, there is a distinct high at the scale level of 4 (corresponding to a time scale of $2^{-4-(-15)} = 2048~\hbox {days} \approx 5.6~\hbox {years}$)—Recall that this is roughly the sampling interval we used in choosing the time-delay series to form the ${\nu }$-dimensional system and compute the information flow. After that period, the sun becomes quiet, correspondingly we do not see much El Niño Modoki for the decade 2010–2020. But two peaks of SSN are seen in the twenty years since 1975–1978. Does this correspondence herald that, after entering 2020, in the following two decades, El Niño Modoki may frequent the equatorial Pacific again? We don’t know whether this indeed points to a link, but it for sure worths an examination. This question, among others, are to be addressed in future studies. (For reference, the codes used in this study are available at http://www.ncoads.org/upload/enso_modoki_data_codes.tar.gz, or https://github.com/EasyCan1994/ENSO.)

## Method

### Estimation of information flow and causality among multivariate time series

Causal inference is of central importance in scientific research^43^. During the past few years it has attracted enormous interest as its role in AI gradually comes to be known. Causal inference is traditionally formulated as a statistical testing problem ever since Granger^44^. Recently, however, it has been recognized that causality is actually a real physical notion attached to information flow, and can be rigorously derived from first principles^20^. Information flow is a concept in general physics which has wide applications. Its importance lies beyond the literal meaning in that it implies causation, and, moreover, provides a quantitative measure of causation. Though with a history of research for more than 40 years, it has just been rigorously formulated, initially motivated by the predictability study in atmosphere-ocean science^19^. Since its birth it has been validated with many benchmark dynamical systems such as baker transformation, Hénon map, Rössler system, etc. (cf.^20,40^), and has been applied with success to different problems in earth system science, neuroscience and quantitative finance, e.g.,^22–24^, to name a few. Hereafter we just give a very brief introduction.

Consider a dynamical system

2 $$\begin{aligned} \frac{d \mathbf{x}}{d t} = \mathbf{F}(t; \mathbf{x}) + \mathbf{B}(t; \mathbf{x}) \dot{\mathbf{w}}, \end{aligned}$$

where $\mathbf{x}$ and $\mathbf{F}$ are ${\nu }$-dimensional vector, $\mathbf{B}$ is a ${\nu }$-by-*m* matrix, and $\dot{\mathbf{w}}$ an *m*-vector of white noise. Here we follow the convention in physics and do not distinguish the notation of a random variable from that of a deterministic variable. Liang^20^ established that the rate of uncertainty (in terms of Shannon entropy) transferred from $x_2$ to $x_1$, or rate of information flowing from $x_2$ to $x_1$ (in nats per unit time), is:3where *E* stands for mathematical expectation, $\rho _1 = \rho _1(x_1)$ is the marginal probability density function (pdf) of $x_1$, $\rho$ , and $g_{11} = \sum _{j=1}^m b_{1j} b_{1j}$. The units are in nats per unit time. Ideally if $T_{2\rightarrow 1} = 0$, then $x_2$ is not causal to $x_1$; otherwise it is causal (for either positive or negative information flow). But in practice significance test is needed.

Generally $T_{2\rightarrow 1}$ depends on $(x_3,\ldots ,x_{\nu })$ as well as $(x_1, x_2)$. But it has been established that^21^ *it is invariant upon arbitrary nonlinear transformation of* $(x_3,x_4,\ldots ,x_{\nu })$, indicating that information flow is an intrinsic physical property. Also established is the *principle of nil causality*, which asserts that, if the evolution of $x_1$ is independent of $x_2$, then $T_{2\rightarrow 1}=0$. This is a quantitative fact that all causality analyses try to verify in applications, while in the framework of information flow it is a proven theorem.

In the case of linear systems where $\mathbf{F}(\mathbf{x},t) = \mathbf{A} \mathbf{x}$, $\mathbf{A} = (a_{ij})$, the formula (3) becomes quite simple^20^:

4 $$\begin{aligned} T_{2\rightarrow 1} = a_{12} \frac{\sigma _{12}}{\sigma _{11}}, \end{aligned}$$

where $\sigma _{ij}$ is the population covariance. An immediate corollary is that *causation implies correlation, while correlation does not imply causation*, fixing the long-standing philosophical debate over causation versus correlation ever since Berkeley^45^.

To arrive at a practically applicable formula, we need to estimate (4), given ${\nu }$ observational time series. The estimation roughly follows that of Liang^40^, and can be found in^46^. The following is just a brief summary of some relevant pieces as detailed in^46^.

Suppose we have ${\nu }$ time series, $x_i$, $i=1,\ldots ,{\nu }$, and these series are equi-spaced, all having *N* data points $x_i(n)$, $n=1,2,\ldots ,N$. Assume a linear model $\mathbf{F}(\mathbf{x};t) = \mathbf{A} \mathbf{x}$, $\mathbf{A} = (a_{ij})$ being a ${\nu }\times {\nu }$ matrix), and $\mathbf{B}$ being a ${\nu }\times {\nu }$ diagonal matrix. To estimate $T_{2\rightarrow 1}$, we first need to estimate $a_{12}$. As shown before in^40^, when $a_{ij}$ and $b_i$ are constant, the maximal likelihood estimator (mle) is precisely the least square solution of the following *N* (overdetermined) algebraic equations

5 $$\begin{aligned} \sum _{j=1}^{\nu }a_{1j} x_j(n) = \dot{x}_1(n),\qquad n=1,\ldots ,N \end{aligned}$$

where $\dot{x}_1(n) = (x_1(n+1) - x_1(n)) / \Delta t$ is the differencing approximation of $dx_1/dt$ using the Euler forward scheme, and $\Delta t$ is the time stepsize (not essential; only affect the units). Following the procedure in^40^, the least square solution of $(a_{11},\ldots , a_{1{\nu }})$, $({{\hat{a}}}_{11},\ldots , {{\hat{a}}}_{1{\nu }})$, satisfies the algebraic equation

$$\begin{aligned} \left[ \begin{array}{ccc} C_{1,1} &{}... &{}C_{1,{\nu }} \\ \vdots &{}\vdots &{}\vdots \\ C_{{\nu },1} &{}... &{}C_{{\nu },{\nu }} \end{array}\right] \left[ \begin{array}{c} {{\hat{a}}}_{11} \\ \vdots \\ {{\hat{a}}}_{1{\nu }} \end{array}\right] = \left[ \begin{array}{c} C_{1,d1} \\ \vdots \\ C_{{\nu },d1} \end{array}\right] , \end{aligned}$$

where

$$\begin{aligned} C_{ij}= & {} \frac{1}{N} \sum _{n=1}^N (x_i(n) - {{\bar{x}}}_i) (x_j(n) - {{\bar{x}}}_j) \\ C_{i,dj}= & {} \frac{1}{N} \sum _{n=1}^N (x_i(n) - {{\bar{x}}}_i) (\dot{x}_j(n) - {\bar{\dot{x}}}_j) \end{aligned}$$

are the sample covariances. Hence ${{\hat{a}}}_{12} = \frac{1}{\det \mathbf{C}} \cdot \sum _{j=1}^{\nu }\Delta _{2j} C_{j,d1}$ where $\Delta _{ij}$ are the cofactors. This yields an estimator of the information flow from $x_2$ to $x_1$:

6 $$\begin{aligned} {{\hat{T}}}_{2\rightarrow 1} = \frac{1}{\det \mathbf{C}} \cdot \sum _{j=1}^{\nu }\Delta _{2j} C_{j,d1} \cdot \frac{C_{12}}{C_{11}}, \end{aligned}$$

i.e., Eq. (6) in “Method”. Here $\det \mathbf{C}$ is the determinant of the covariance matrix $\mathbf{C}$, and now *T* is understood as the MLE of *T*. We slightly abuse notation for the sake of simplicity.) For two-dimensional (2D) systems, ${\nu }=2$, the equation reduces to

7 $$\begin{aligned} {{\hat{T}}}_{2\rightarrow 1} = \frac{C_{11}C_{12} C_{2,d1} - C_{12}^2 C_{1,d1}}{C_{11}^2 C_{22} - C_{11}C_{12}^2}, \end{aligned}$$

recovering the familiar one as obtained in^40^ and frequently used in applications (e.g.,^22–24^).

### Significance test

The significance of ${{\hat{T}}}_{2\rightarrow 1}$ in (6) can be tested following the same strategy as that used in^40^. By the MLE property (cf.^47^, when *N* is large, ${{\hat{T}}}_{2\rightarrow 1}$ approximately follows a Gaussian around its true value with a variance $\left( \frac{C_{12}}{C_{11}}\right) ^2 {{{\hat{\sigma }}}}_{a_{12}}^2$. Here ${{{\hat{\sigma }}}}_{a_{12}}^2$ is the variance of ${{\hat{a}}}_{12}$, which is estimated as follows. Denote by ${\varvec{\theta }}$ the vector of parameters to be estimated; here it is $(a_{11}, a_{12}, a_{13},\ldots ,a_{1{\nu }}; b_1)$. Compute the Fisher information matrix $\mathbf{I} = (I_{ij})$

$$\begin{aligned} I_{ij} = - \frac{1}{N} \sum _{n=1}^N \frac{\partial ^2 \log \rho (\mathbf{x}_{n+1} | \mathbf{x}_n; \hat{{\varvec{\theta }}} ) }{\partial \theta _i \partial \theta _j} \end{aligned}$$

where $\rho (\mathbf{x}_{n+1} | \mathbf{x}_n)$ is a Gaussian for a linear model:

$$\begin{aligned} \rho (\mathbf{x}_{n+1} | \mathbf{x}_n)= & {} \frac{1}{[(2\pi )^2\det (\mathbf{B} \mathbf{B}^T \Delta t )]^{1/2}} \\&\times e^{-\frac{1}{2} (\mathbf{x}_{n+1}-\mathbf{x}_n -\mathbf{A}\mathbf{x}_n\Delta t)^T (\mathbf{B}\mathbf{B}^T\Delta t)^{-1} (\mathbf{x}_{n+1}-\mathbf{x}_n -\mathbf{A}\mathbf{x}_n\Delta t) }. \end{aligned}$$

The computed entries are then evaluated using the estimated parameters, and hence the Fisher information matrix is obtained. It has been established (cf.^47^) that $\mathbf{I}^{-1}/N$ is the covariance matrix of $\hat{{\varvec{\theta }}}$, from which it is easy to find the entry ${\hat{\sigma }}_{a_{12}}^2$ [here it is the entry (2,2)]. Given a level, the significance of an estimated information flow can be tested henceforth.

More robust testing requires that surrogate data be generated for estimating the background noise of observed data^48^. Autoregressive (AR) surrogates provide typical realizations; particularly, red noise can be perfectly described by the AR model of the first order, written AR(1), while a large portion of climate data has a red noise background. See the comprehensive review^48^ and the references therein. We hence adopt for our purpose the following model

8 $$\begin{aligned} x(n) = a + \gamma x(n-1) + b \epsilon (n) \end{aligned}$$

where $\epsilon (n)$ is a standard Gaussian random variable, and *a*, $\gamma$, and *b* are parameters. By the algorithm in Lancaster et al.^48^, with the AR(1) noise assumption, surrogates for a given series are generated via two steps: (1) fit the series to the model to obtain the parameters as mentioned above; (2) produce a set of surrogate series by solving (8) with the estimated *a*, $\gamma$ and *b*, and a set of random initial conditions.

The null hypothesis is that the given time series can be fully described by the above AR(1) noise model, which implies that, for two series, there is no causality between the respective surrogates of them. To reject the null hypothesis, the information flows computed with the observational series should lie beyond the preset percentile of the information flows based on the surrogates. Usually one may choose a 90th percentile, a 95th percentile, or a 99th percentile.

The number of surrogates, written *M*, makes an issue in this study, as there are more than 10,000 time series within the study domain. According to^48^, $M = 2K/\alpha - 1$ surrogates are needed for a two-sided test. Here *K* is some integer and $\alpha$ the significance level. So for $K=1$, $\alpha =0.05$, $M = 39$. In this study we have tried $M=100$ and $M=200$, and the results are similar.

## Supplementary information

Supplementary Information.

## Data Availability

The data used in this study include the SST and sunspot number (SSN) from National Oceanic and Atmospheric Administration (NOAA) (SST: http://www.esrl.noaa.gov/psd/data/gridded/cobe2.html; SSN: https://wwww.esrl.noaa.gov/psd/gcos_wgsp/Timeseries/Data/sunspot.long.data), and the El Niño Modoki Index (EMI) from Japan Agency for Marine-Earth Science and Technology (JAMSTEC) (http://www.jamstec.go.jp/aplinfo/sintexf/e/elnmodoki/data.html). Among them SST has a horizontal resolution of $1^{{\text{o}}}\times 1^{{\text{o}}}$. Temporally all of them are monthly data, and at the time when this study was initiated, SST, EMI, and SSN respectively have a time coverage of 01/1850–12/2018, 01/1870–11/2018, and 05/1847–12/2017. Daily SSN is also used for spectral analysis; it covers the period 15/04/1921–31/12/2010.

## Code availability

All the codes (and some of the downloaded data) are available at http://www.ncoads.org/upload/enso_modoki_data_codes.tar.gz, or https://github.com/EasyCan1994/ENSO.
