# Supplementary material for: El Niño Modoki can be mostly predicted more than 10 years ahead of time
Source: Sci Rep. 2021 Sep 9;11:17860. doi: 10.1038/s41598-021-97111-y (PMC8429568; doi:10.1038/s41598-021-97111-y)
Supplement: Supplementary file 1 — Supplementary Information. [file 41598_2021_97111_MOESM1_ESM.docx]

**El Niño Modoki can be mostly predicted more than 10 years ahead of time**

X. San Liang^1,2,3,4*^, Fen Xu^2^, Yineng Rong^2^,

Renhe Zhang^1,3,4^, Xu Tang^1,4^, Feng Zhang^1,3^

^1^Department of Atmospheric and Oceanic Sciences, and Institute of Atmospheric Sciences, Fudan University, Shanghai 200438, China

^2^Nanjing Center for Ocean-Atmosphere Dynamical Studies, Nanjing Institute of Meteorology, Nanjing 210044, China

^3^Shanghai Qi Zhi Institute (Andrew C. Yao Institute), Shanghai 200232, China

^4^IRDR ICoE on Risk Interconnectivity and Governance on Weather/Climate Extremes Impact and Public Health, Fudan University, Shanghai 200438, China

***Corresponding author:**

X. S. Liang

Dept. Atmos. & Oceanic Sci., Fudan University

No. 2005 Songhu Rd, Yangpu District, Shanghai 200438, China

Email: sanliang@courant.nyu.edu

URL: http://www.ncoads.org/

**Supplementary information**

**Supplementary Figure S1:** The absolute information flow estimated using the bivariate formula from the delayed series of sunspot numbers (SSN) to those of the sea surface temperature (SST) in the Pacific Ocean (in nats/month). The SST time series between January 1980 – December 2017 are used. In each subplot, the lower panel shows only the value that is significant at a 90% confidence level, and indicated on it is the delay (in years). (Figure generated with MATLAB, Version 6.5. http://www.mathworks.com/.)

**Supplementary Figure S2:** The lower bound (a) and upper bound (b) at the 99^th^ percentile of the information flows (in nats/month; using the multivariate formula) from the surrogates of the SSN series delayed by 45 years to the surrogates of the SST series. The surrogates are generated with the first order autoregressive [AR(1)] noise model based on the SST time series between January 1980 – December 2017.

(Figure generated with MATLAB, Version 6.5. http://www.mathworks.com/.)

**Supplementary Figure S3:** As Figure 1b in the text, but the significance test is performed using surrogates generated with the AR(1) noise model. Top: the confidence level is 90%. Bottom: the confidence level is 99%. (Figure generated with MATLAB, Version 6.5. http://www.mathworks.com/.)

**Supplementary Figure S4:** As Supplementary Figure S1, but the information flows are estimated using the multivariate formula, with the embedding coordinates formed with SSN series lagged by 22-50 years every 5 years. (Figure generated with MATLAB, Version 6.5. http://www.mathworks.com/.)

**Supplementary Figure S5.** The absolute information flow from the SSN series delayed by 45 years to those of the SST in the Pacific Ocean (in nats/month). It is estimated with the multivariate formula using embedding coordinates formed with SSN series delayed by 22-50 years every 5 years. The SST time series between January 1980 – December 2005 are used. In each subplot, the lower panel shows only the value that is significant at a 90% confidence level. (Figure generated with MATLAB, Version 6.5. http://www.mathworks.com/.)

**
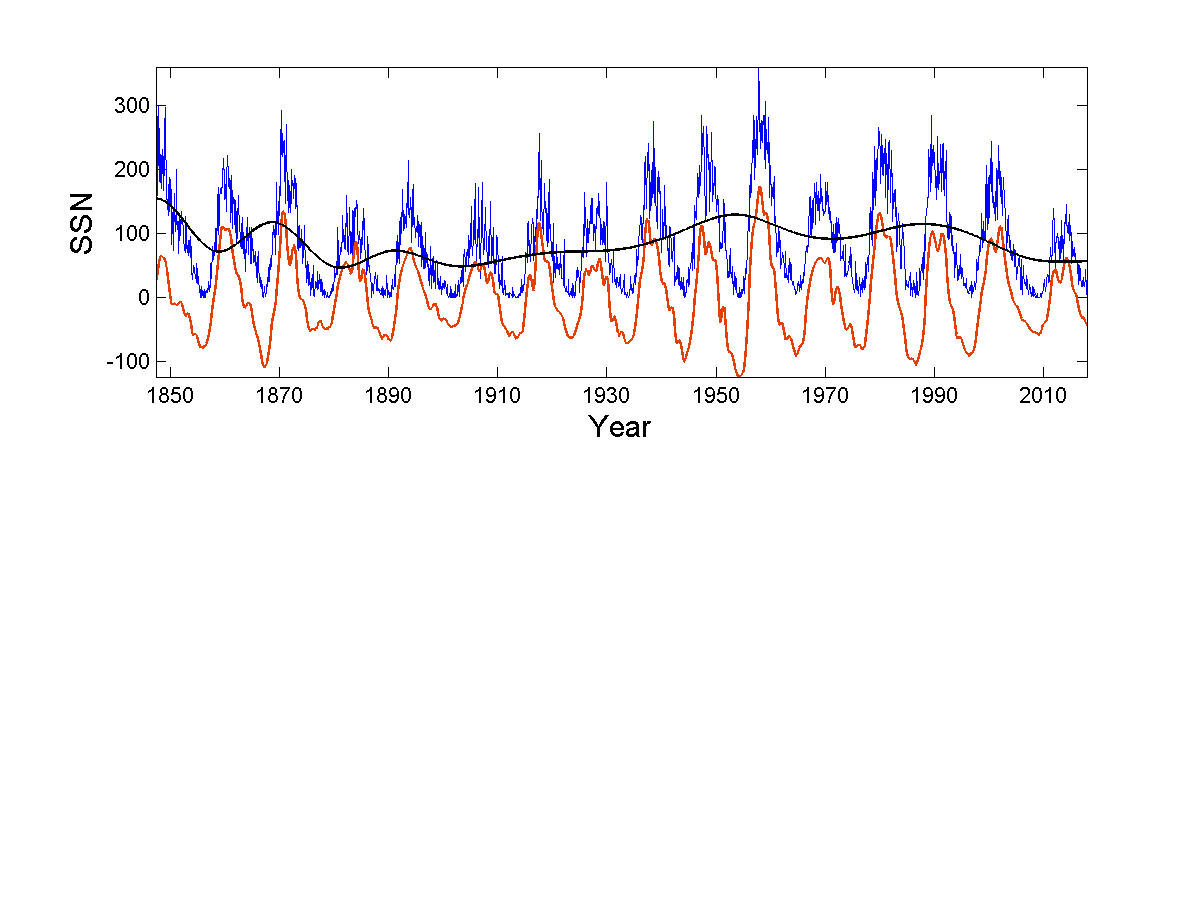
**

**Supplementary Figure S6.** The original monthly SSN series (blue), lowpass-filtered series (black), and bandpass-filtered series (red). (Figure generated with MATLAB, Version 6.5. http://www.mathworks.com/.)

**Supplementary Figure S7.** Projection of the El Niño Modoki Index (EMI) based solely on the SSN 22-50 years ago with a simple linear regression model. Upper: The SSN series as input is not filtered. Lower: The SSN series is low-pass filtered. The observed EMI is in blue, while the projected is in red. (Figure generated with MATLAB, Version 6.5. http://www.mathworks.com/.)

**Supplementary Figure S8.** Normalized variance vs. EOF mode. The EOF analysis is performed with the SSN time series with delays from 22-50 years, which form a vector with 336 members of series. (Figure generated with MATLAB, Version 6.5. http://www.mathworks.com/.)

**Supplementary Figure S9.** Examples of the EOF modes as described in Supplementary Figure S8. (Figure generated with MATLAB, Version 6.5. http://www.mathworks.com/.)

**Supplementary Figure S10.** As Figure 2b, but the projection uses, respectively, the first 8 and 50 principal components as inputs. (Figure generated with MATLAB, Version 6.5. http://www.mathworks.com/.)

**
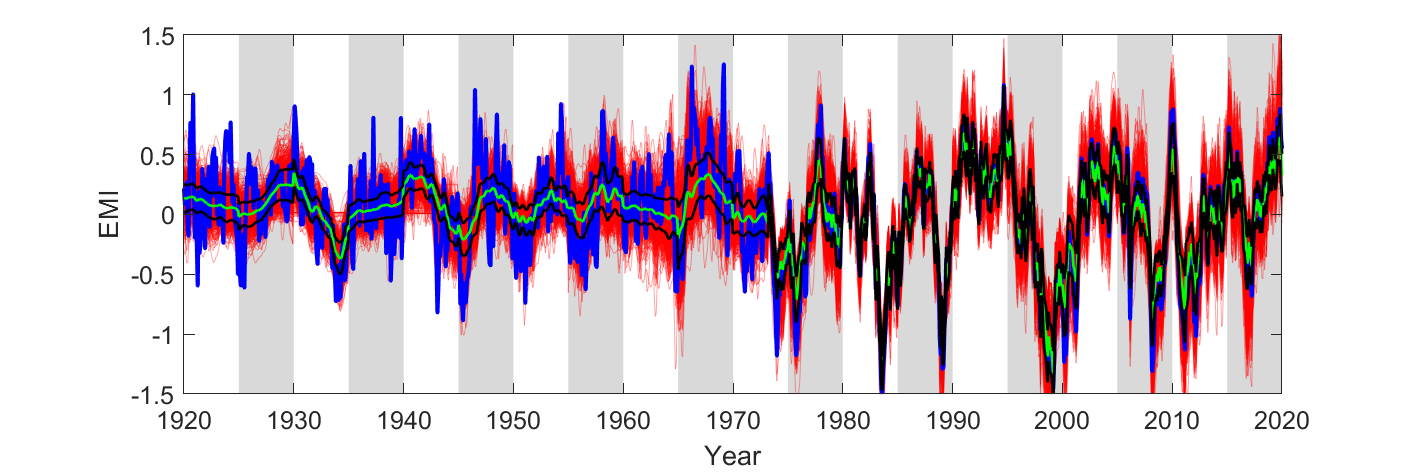
**

**
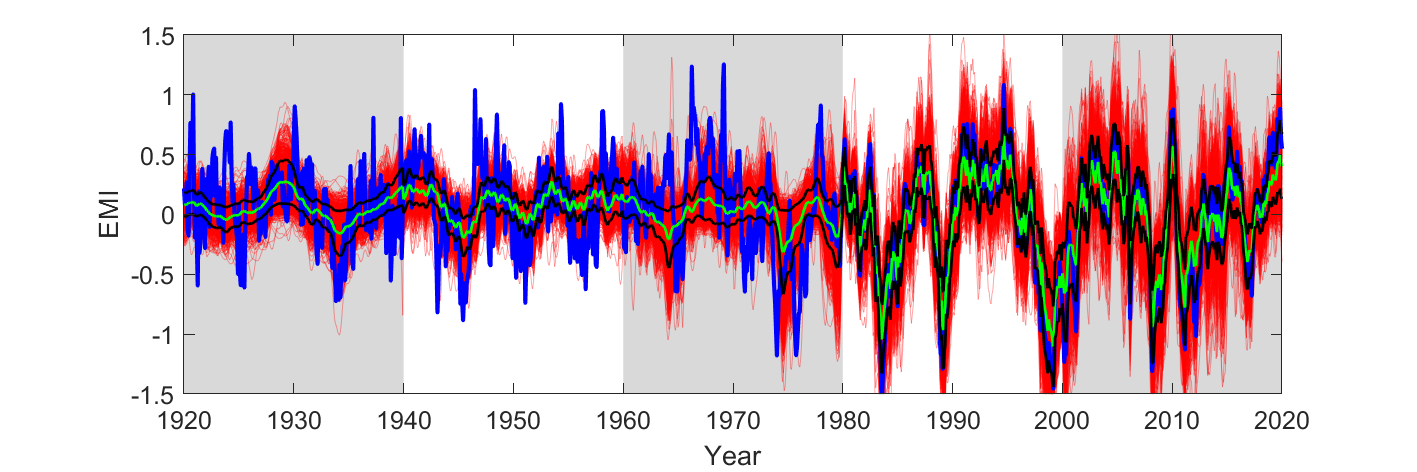
**

**Supplementary Figure S11.** Results of cross-validations. For a validation period, use the rest data to train the model, then swap the interval until every period has been utilized for validation once. The “forecasts” for all the validation periods are then pieced together for form the plot. The observed EMI is in blue, the ensemble mean of the predicted EMIs is in green, and the black lines demarcate the standard deviation. Upper: the validation period is 5 years. Lower: the validation period is 20 years. Note, as explained in the text, for a dissipative system such as the atmosphere/ocean, future cannot be used to “predict” the past (“arrow of time”). This accounts for the performance of the cross-validations before 1980 as shown in the figure.

(Courtesy of an anonymous reviewer. Figures generated with MATLAB, Version 6.5. http://www.mathworks.com/.)

**(a)**

**
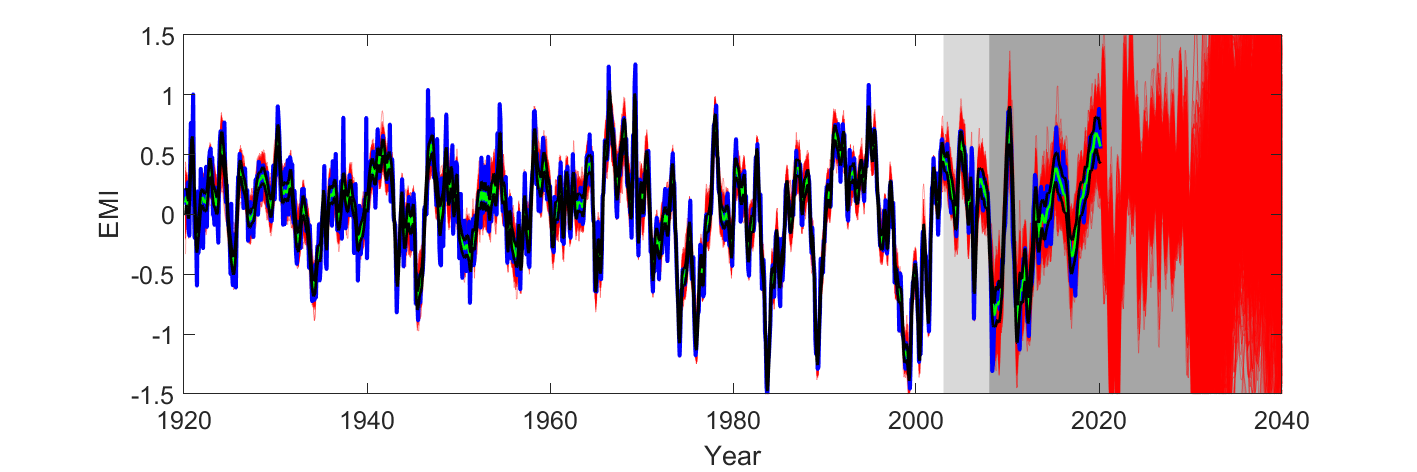
**

**(b)**

**Supplementary Figure S12.** (a) The 1000 predictions of EMI with the originally trained model into the future (as Figure 5 but with forecasts through 2039). (b) The standard deviation of the predictions. (Figure generated with MATLAB, Version 6.5. http://www.mathworks.com/.)

**Supplementary Figure S13.** The bivariate information flow from SSN to SST lagged by 45 years in each decade from 1950 to 2020. (Figure generated with MATLAB, Version 6.5. http://www.mathworks.com/.)

**Supplementary Figure S14.** The bivariate information flow from SSN to SST lagged by 10 years (left) and 43 years (right) from 2010 to 2020. (Figure generated with MATLAB, Version 6.5. http://www.mathworks.com/.)
